# Comparison of intragastric pressure between endotracheal tube and supraglottic airway devices in laparoscopic hepatectomy: A randomized, controlled, non-inferiority study

**DOI:** 10.1097/MD.0000000000026287

**Published:** 2021-06-18

**Authors:** Jin Hee Ahn, Ji Seon Jeong, Se Hee Kang, Ji Eun Yeon, Eun A. Cho, Gyu Sung Choi, Jong Man Kim, Gaab Soo Kim

**Affiliations:** a Department of Anaesthesiology and Pain Medicine, Kangbuk Samsung Hospital; b Department of Anaesthesiology and Pain Medicine, Samsung Medical Center, Sungkyunkwan University School of Medicine; c Department of Anaesthesiology and Pain Medicine, Department of Anaesthesiology and Pain Medicine, CHA University Ilsan Medical Center, College of Medicine, CHA University of Korea; d Department of General Surgery, Samsung Medical Center, Sungkyunkwan University School of Medicine, Seoul, Republic of Korea.

**Keywords:** airway management, hepatectomy, laparoscopic surgery, laryngeal mask, supraglottic device

## Abstract

**Background:**

Supraglottic airway (SGA) devices do not definitively protect the airway from regurgitation of gastric contents. Increased gastric pressure and long operation time are associated with development of complications such as aspiration pneumonia. The aim of this study was to compare intragastric pressure between second-generation SGA and endotracheal tube (ETT) devices during long-duration laparoscopic hepatectomy.

**Methods:**

A total of 66 patients was randomly assigned to 2 groups; 33 patients each in the ETT and SGA groups. Intragastric pressure was continuously measured via a gastric drainage tube with a three-way stopcock connected to the pressure monitoring device. Normal saline was added to the end of the gastric drainage tube at each operation time point.

**Results:**

Intragastric pressure during pneumoperitoneum was no different between the 2 groups (*P* = .146) or over time (*P* = .094). The mean (standard deviation [SD]) pH of the SGA tip measured after operation was 6.7 (0.4), and a pH <4 was not observed. Relative risk of postoperative complications was significantly higher in the ETT group relative to the SGA group (sore throat, 5.5; cough,13.0).

**Conclusions:**

Use of SGA devices does not further increase intragastric pressure, even during prolonged upper abdominal laparoscopic surgery. Also, the frequency of postoperative sore throat and cough was significantly lower when the second-generation SGA device was used.

## 1 Introduction

Supraglottic airway (SGA) devices can be used as an alternative for endotracheal tube (ETT) during general anesthesia.^[1]^ SGAs are quick and easy to use and have a lower incidence of postoperative complications such as sore throat, dysphagia, and hoarseness.^[2,3]^ However, SGAs do not offer definitive airway protection from regurgitation of gastric contents.^[3]^

Increased gastric pressure is associated with development of complications such as aspiration pneumonia.^[4]^ In particular, intragastric pressure increases in laparoscopic surgery which can increase the likelihood of gastroesophageal reflux with SGA.^[5,6]^ In previous studies, intragastric pressure and visually scaled gastric distension score were not different between SGA and ETT during laparoscopic surgery.^[7]^ However, previous studies are limited to pediatric surgery with short operating times, use subjective indicators, or use first-generation SGA (classic laryngeal mask airway). Long operation times increase the incidence of regurgitation of gastric contents.^[8]^ On the other hand, second-generation SGAs with gastric drainage ports improve safety of aspiration.^[9]^ However, no studies have addressed the safety of second-generation SGAs in major abdominal surgery with long operation time under laparoscopy.

Therefore, we conducted the present study to compare the intragastric pressures between second-generation SGA and ETT during long-duration laparoscopic hepatectomy. The primary outcome was difference in intragastric pressure between the second-generation SGA and ETT. The secondary outcome was difference in postoperative complications.

## 2 Methods

### 2.1 Ethics and study design

**Figure 1:**
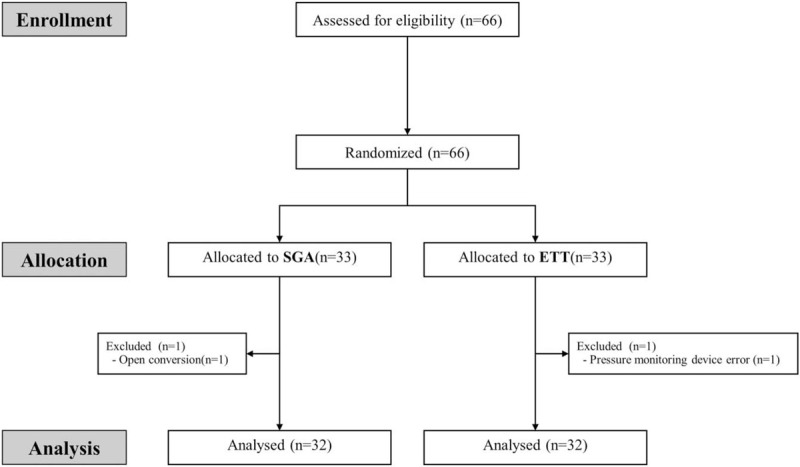
Consolidated standards of reporting trials flow diagram.

This prospective study was performed at Samsung Medical Center in Seoul, Korea, after approval from Samsung Medical Center Institutional Review Board (IRB No. 2018-10-111). This trial was registered at Clinical Trials of Korea (KCT 0003512). This study was retrospectively registered (February 15, 2019) after enrollment of the first participant (February 04, 2019). Patients with an American Society of Anaesthesiologists physical status of I–III and who were scheduled for elective laparoscopic hepatectomy were enrolled between February 2019 and March 2019. Patients were excluded if they had lung disease, upper respiratory infection symptoms, esophageal varix, or previous stomach surgery. Written informed consent was obtained from all participants. This manuscript adhered to the Consolidated Standards of Reporting Trials guidelines (Fig. 1).

### 2.2 Blinding and randomization

All patients were randomly assigned to one of 2 groups using a randomization list administered by a nurse who did not participate in the study. A sealed envelope with group assignment was given to the investigator just before the operation. One experienced investigator performed the airway management, and the intraoperative data were recorded by those who did not participate in airway management.

### 2.3 Anesthesia protocol

All patients underwent midnight Nil per Os. Standard monitoring such as electrocardiography, pulse oximetry, noninvasive blood pressure, and bispectral index monitoring were performed on arrival at the operating room. Anesthesia was induced using 5 mg kg^−1^ of thiopental sodium and 8 vol% of sevoflurane. After loss of consciousness (LOC) was confirmed, 0.8 mg kg^−1^ rocuronium was administered. Mask ventilation was performed such that the airway pressure did not exceed 20 cm H_2_O. Endotracheal intubation or SGA insertion was performed after confirming maximum neuromuscular blockade with twice Train-of-Four (TOF) count 0. And, the surgical procedure was performed in a deep neuromuscular block (no responses to TOF and 2 or fewer responses to post-tetanic count). Endotracheal tube (Shiley, Hi-Contour Oral Tracheal Tube Cuffed, Covidien, Germany) intubation (Group ETT) or second-generation SGA (LMA Protector, Teleflex Medical Ltd., Athlone, Ireland) insertion (Group SGA) was performed. Mask ventilation was performed such that the airway pressure did not exceed 20 cm H_2_O. The SGA size was selected according to manufacturer recommendations. Air was insufflated into the SGA cuff until the pilot balloon black line was located within the green zone.^[10]^ After successful ventilation was confirmed on capnography, mechanical ventilation was started. Cuff pressure of the SGA was adjusted using a digital cuff pressure monitor (Shiley Pressure Control, Covidien, Germany) to maintain ≤60 cm H_2_O. SGA devices were replaced by ETTs in the following situations for patient safety if the SGA was not inserted in >3 attempts, persistent oropharyngeal leak with inadequate ventilation (end-tidal CO_2_ ≥45 mm Hg during pneumoperitoneum), and/or the stomach was inflated enough to cause visual disturbance under laparoscopic view. If the SGA was replaced by ETT, the patient was dropped from the study. The ETT size was selected according to sex (7.0 mm for women, 8.0 mm for men). The ETT cuff pressure was monitored using a digital cuff pressure monitor to ensure that it did not exceed 25 cm H_2_O.^[11,12]^

Tidal volume was 8 mL kg^−1^ (ideal body weight), and respiratory rate was adjusted as required to maintain end-tidal CO_2_ at 35 to 40 mm Hg. Tidal volume was reduced if airway pressure exceeded 25 cm H_2_O. After ETT intubation or SGA insertion, a non-compressible 14 French gastric drainage catheter (ST probe, Lucky Medical Co., LTD, Seoul, Korea) was inserted and fixed up to 60 cm through the mouth or the SGA gastric drainage port. After anesthesia induction, patient posture was adjusted to the French position by surgeon request. The surgeon inserted a trocar into the peritoneal cavity, and the CO_2_ insufflator was maintained to 12 mm Hg during pneumoperitoneum. After operation, the gastric drainage catheter was sufficiently suctioned and carefully removed.

### 2.4 Intragastric pressure monitoring and data acquisition

**Figure 2:**
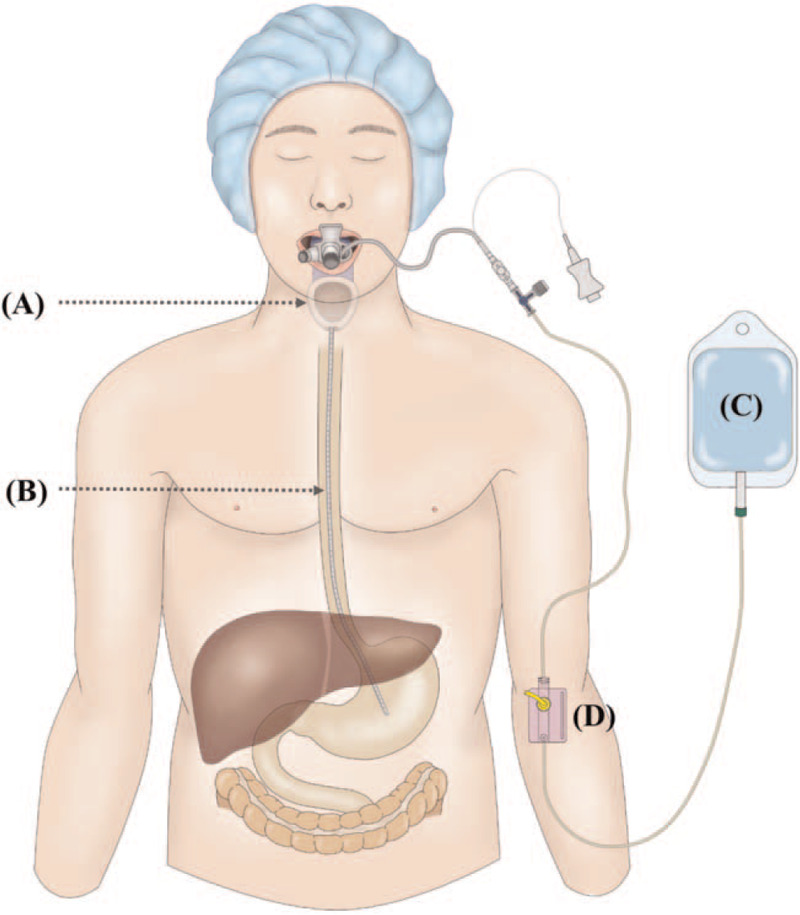
Schematic diagram of intragastric pressure measurement. (A) LMA Protector, (B) gastric drainage catheter, (C) normal saline with pressure bag, (D) pressure monitoring sensor. (^∗^This image is author's own work, freely available to use.).

After gastric drainage catheter insertion, sufficient intragastric suction was performed. The surgeon confirmed that the gastric drainage catheter tip was located on the stomach. The gastric drainage catheter with a 3-way stopcock was connected to the pressure monitoring device and filled with normal saline. Intragastric pressure was continuously measured by a pressure monitoring device (Edward Life Science, TruWave 3 cm^3^/60 in; Fig. 2). The pressure transducer was calibrated and placed at the intersection of the anterior axillary line and a transverse line at the level of the xyphoid.^[13]^ In SGA, the airway sealing pressure was controlled by a pressure value that reached equilibrium at a fresh gas flow of 3 L min^−1^ with the adjustable pressure limiting valve closed.^[14]^ If the sealing pressure increased above 30 cm H_2_O, we stopped the measurement. Intragastric pressure was collected at several time points (baseline, T1–T6 during pneumoperitoneum every 30 minutes, and at the end of surgery) during the anesthesia period by the monitoring computer program (Picis Care Suit Anaesthesia Manager; Picis Ltd., Wakefield, MA). The pH was measured after removing the SGA by sampling secretions on the tip of the SGA using a spatula and placing the drop at the tip of a pH meter (2K712, ISFETCOM Co., Ltd., Japan). Total anesthesia time, total operation time, and total pneumoperitoneum time were recorded. Postoperative complications such as sore throat, cough, hoarseness, and laryngospasm were assessed from the time immediately after removal of the airway device to discharge from the post anesthesia care unit.

### 2.5 Statistics

In an initial pilot study, sample size calculations were performed using a non-inferiority test with Student *t* test to identify differences between intragastric baseline and peak pressures in ETT and SGA. Changes of intragastric pressure during surgery were 14.8 mm Hg in ETT and 14 mm Hg in SGA (standard deviation 2.8, non-inferiority margin 3.0). The sample size was calculated with a power of 0.9 and an alpha error of 0.05. Assuming a 10% dropout rate, we planned to recruit a total of 66 patients (33 subjects for each group).

The change of difference between intragastric baseline and peak pressures in ETT and SGA was calculated with a 95% confidence interval (CI). If the lower limit of the 95% CI was <3 mm Hg, non-inferiority of SGA compared with ETT was demonstrated. Continuous variables are presented as the mean ± standard deviation (SD) or median (interquartile range, IQR) as appropriate. Continuous variables were compared using Student *t* test or Wilcoxon signed-rank test, and the Shapiro-Wilk test was used to explore normality. Categorical variables were analyzed using Pearson chi-square test or Fisher exact test as appropriate. The difference between baseline and each time point of intragastric pressure between the 2 groups was analyzed using generalized estimating equations (GEE). Bonferroni correction for post-hoc analysis was applied. Statistical analyses were performed using SPSS version 22 (SPSS Inc., Chicago, IL). A *P*-value <.05 was considered statistically significant.

## 3 Results

In this prospective randomized study, a total of 66 patients was enrolled, of which 33 were included in each the ETT and SGA groups. One patient in the SGA was excluded due to open conversion during operation, and 1 patient in the ETT was excluded due to pressure monitoring device error. Therefore, a total of 64 patients was analyzed.

**Figure 3:**
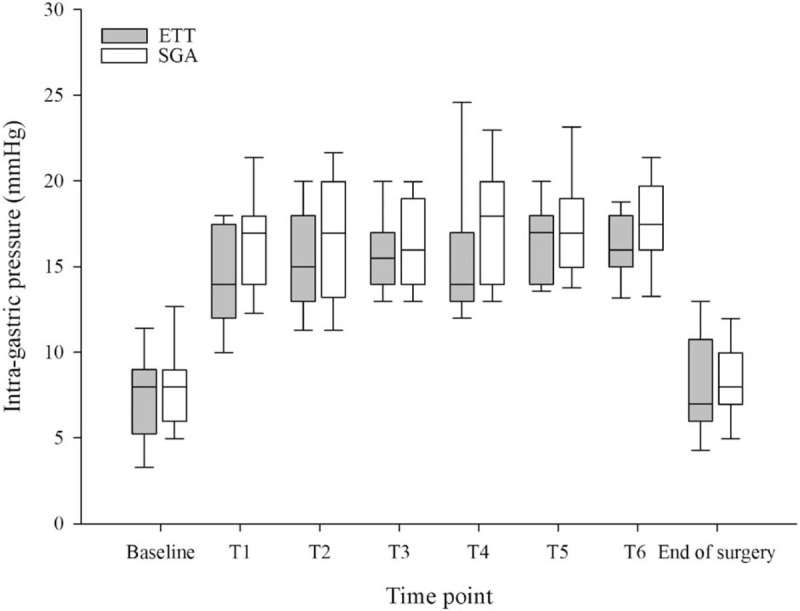
Intragastric pressure at each time point. T1–T6, during pneumoperitoneum every 30 minutes.

There were no significant differences in patient characteristics and surgery/anesthesia data (Table 1). The difference between intragastric baseline and peak pressure was 10.63 mm Hg in the ETT and 11.81 mm Hg in the SGA (mean difference, –1.188 mm Hg; 95% CI, –2.845–0.470 mm Hg; *P* = .157). The lower limit of the 95% CI was <3 mm Hg, demonstrating non-inferiority of SGA compared with ETT. However, superiority was not significant. The intragastric pressure at each time point is shown in Fig. 3. The intragastric pressure during pneumoperitoneum was no different between the 2 groups (*P* = .146) or over time (*P* = .094). The mean (SD) intragastric pressure at baseline and at the end of surgery were 7.3 (2.8) and 8.3 (3.6) mm Hg in ETT (mean difference, 0.9 mm Hg; 95% CI, 0–1.9 mm Hg; *P* = .047), respectively, and 8.1 (2.6) and 8.4 (2.9) mm Hg in SGA (mean difference, 0.4 mm Hg; 95% CI, –0.8–1.6 mm Hg; *P* = .544). The mean (SD) pH of the SGA tip measured after operation was 6.7 (0.4), and a pH <4 was not observed. The median (IQR) of SGA leakage pressure was 25 (21–30) mm Hg immediately after anesthesia induction and 24 (20–29) mm Hg after pneumoperitoneum.

**Table 1:** Patient characteristics and surgery/anesthesia data.

	SGA (n = 32)	ETT (n = 32)
Sex (female/male)	9/23	10/22
Age, mo	55.1 (11.3)	59.0 (9.7)
Weight, kg	66.1 (11.9)	65.0 (12.0)
Height, cm	164.0 (6.0)	164.8 (8.3)
BMI	24.4 (3.2)	23.8 (3.1)
ASA PS (I/II/III)	14/16/2	12/18/2
HCC size	2.5 [1.9,6.7]	2.7 [1.7,4.0]
Anesthetic time, min	204 (64)	208 (55)
Operation time, min	159 (62)	153 (56)
Insufflation time, min	129 (51)	124 (54)

Postoperative complications such as sore throat and cough were significantly higher in ETT than in SGA (RR [relative risk] = 5.5, *P* < .001; RR = 13.0, *P* < .001, respectively; Table 2). However, hoarseness was not significantly different between the 2 groups (*P* = .113), and laryngospasm did not occur in either group. There were no cases where SGA was replaced by ETT for patient safety.

**Table 2:** Postoperative airway complications.

	SGA (n = 32)	ETT (n = 32)	RR [95% CI]	*P*-value
Sore throat	4 (13)	22 (69)	5.5 [2.1, 14.2]	<.001
Cough	1 (3)	13 (41)	13.0 [1.8, 93.6]	<.001
Hoarseness	0 (0)	4 (13)	9.0 [0.5, 160.6]	.113
Laryngospasm	0 (0)	0 (0)	-	-

## 4 Discussion

We compared changes in intragastric pressure according to airway device to investigate the changes of intragastric pressure in laparoscopic hepatectomy with long operation time. Our results showed that use of SGA or ETT in laparoscopic hepatectomy did not reveal significant differences in intragastric pressure. In addition, postoperative airway complications such as sore throat and cough were significantly less frequent in SGA.

Pulmonary aspiration is associated with predisposing factors such as delayed gastric empting, no fasting, ileus, pregnancy, and emergency surgery. However, a decrease in lower esophageal tone due to the effects of anesthesia and surgery itself, and reflux of acid with increased intragastric pressure can cause aspiration pneumonitis. Anesthesiologists are reluctant to use SGA in prolonged laparoscopic upper abdominal surgery as prolonged use of SGA increases the risk of pulmonary aspiration.^[8,15–17]^ SGA does not guarantee a complete seal around the larynx, which increases the risk of aspiration pneumonia due to the increase in intragastric pressure.^[18,19]^ Thus, we conducted a study of laparoscopic upper abdominal surgery with long operation time. In our study, mean CO_2_ insufflation time of laparoscopic surgery was 126 minutes; however, intragastric pressure increased about 11 to 13 mm Hg in both groups compared with baseline, and intragastric pressure did not significantly increase in the SGA over time compared with in the ETT. During pneumoperitoneum, intragastric pressure increases, and the risk of aspiration is increased.^[19]^ However, when the lower esophageal sphincter pressure is higher than the intragastric pressure, the risk of aspiration is reduced.^[20]^ Jones et al^[21]^ demonstrated that lower esophageal sphincter pressure increased by 11.6 mm Hg as abdominal pressure increased by 7.7 mm Hg during pneumoperitoneum. This mechanism can reduce the risk of gastroesophageal reflux by increasing esophageal sphincter tone with an adaptive response when intra-abdominal pressure increases. In our study, when we increased the abdominal pressure by 12 mm Hg during pneumoperitoneum, intragastric pressure increased by 11 to 13 mm Hg in both the SGA and ETT groups. Intragastric peak pressure were distributed 17 to 19 mm Hg in both groups. The maximum value of intragastric peak pressure was 29 mm Hg in the ETT group and 25 mm Hg in the SGA group. The pH of the SGA tip measured after surgery was never lower than pH 4, indicating a lack of gastric content aspiration.^[22]^ Although our results suggest no difference in the use of SGA or ETT in long-duration laparoscopic upper abdominal surgery, safety from gastric content aspiration is not assured with SGA.

The incidence of aspiration pneumonitis when using SGA is 0.02%.^[23]^ First-generation SGAs lack design features to reduce the risk of aspiration, while second-generation SGAs offer improved safety against aspiration and regurgitation through separate access to the respiratory tracts and esophageal drainage.^[24]^ The second-generation SGA (Protector) used in our study has special features to increase the safety associated with gastric reflux prevention through a sufficient sealing pressure and suction of gastric contents through a suction port.^[25]^ Thus, second-generation SGAs provide a means of gastric decompression and reduce the gastric distension and the risk of pulmonary aspiration.^[26]^ Use of an ST probe as the gastric pressure measurement device in our study further increased the safety of SGA.

Generally, ETT is related with a greater risk of postoperative sore throat, cough, and/or hoarseness relative to SGA.^[27,28]^ One of the most common complications was sore throat after general anesthesia, with an incidence rate of 62% in a previous study.^[27]^ Although the incidence of postoperative sore throat in SGA is lower than that of ETT, it remains significant at up to 49%.^[29]^ In our study, postoperative sore throat was observed in 69% of the ETT group and in 13% of the SGA group. This disparity is probably attributable to differences in quantifying postoperative sore throat and/or different study methodologies and equipment. Airway complications such as postoperative cough and hoarseness were also lower in SGA than in ETT in this study.

There are several limitations to our study. First, barrier pressure is thought to prevent gastroesophageal reflux and is measured by subtracting intragastric pressure based on lower esophageal sphincter pressure.^[20]^ Since we did not measure low esophageal sphincter pressure, only gastric pressure, we cannot completely rule out lower esophageal sphicter release under general anesthesia condition, one of the causes of gastroesophageal reflux. Therefore, further studies are recommended to confirm barrier pressure. Second, the relationship of intragastric pressure with pneumoperitoneum duration could not be clearly confirmed. The proportion of patients with duration of pneumoperitoneum >120 minutes was 47% in the ETT group and 53% in the SGA group. There was a limit to obtaining statistically significant results according to time course. Third, we could not be certain that the position of the pressure monitoring transducer was exactly in the stomach. We use an anatomical landmark to locate the transducer of the pressure monitoring device relatively close to the stomach. Fourth, pH of the pharynx was measured only in the SGA group to determine postoperative regurgitation of gastric content. Fifth, we did not measure the grade of gastric distension. Visually assessed gastric distension score is too subjective to evaluate the degree of gastric insufflation consistently. Whereas, our study has the advantage of confirming the degree of gastric insufflation using an objective values of intragastric pressure. Sixth, our study population was mainly ASA class 1–2 and body mass index (BMI) was relatively low, 23 to 24. Depending on the presence of underlying disease or in obesity patients, different results may be obtained. Therefore, it is difficult to apply the study results to all populations.

## 5 Conclusions

The use of SGA does not further increase intragastric pressure, even during prolonged upper abdominal laparoscopic surgery. Also, the frequency of postoperative sore throat and cough was significantly less with SGA. Nevertheless, risk of pulmonary aspiration cannot be eliminated, but continued use of a gastric drainage catheter can be reduced the risk.

## Acknowledgments

The authors would like to thank the Statistics and Data Centre of Samsung Medical Centre.

Image (Fig. 2) were drawing using version of Adobe illustrator cc 2018.

## Author contributions

**Conceptualization:** Jin Hee Ahn, Gaab Soo Kim.

**Data curation:** Jin Hee Ahn, Ji Eun Yeon, Eun A. Cho, Gaab Soo Kim.

**Formal analysis:** Jin Hee Ahn, Eun A. Cho, Gyu Sung Choi.

**Investigation:** Jin Hee Ahn, Se Hee Kang, Gyu Sung Choi.

**Methodology:** Jin Hee Ahn, Eun A. Cho.

**Project administration:** Se Hee Kang, Gyu Sung Choi.

**Resources:** Ji Eun Yeon, Jong Man Kim.

**Software:** Jiseon Jeong, Ji Eun Yeon.

**Supervision:** Jong Man Kim, Gaab Soo Kim.

**Validation:** Jong Man Kim.

**Visualization:** Jong Man Kim.

**Writing – original draft:** Jin Hee Ahn, Jiseon Jeong.

**Writing – review & editing:** Gaab Soo Kim.

## Correction

When originally published, the name Ji Seon Jeong appeared with the incorrect spacing as Jiseon Jeong. “Samsung Medical Center, Sungkyunkwan University School of Medicine” was originally left off of affiliation b and has now been added.
